# Theoretical Approach for Electron Dynamics and Ultrafast Spectroscopy (EDUS)

**DOI:** 10.1021/acs.jctc.2c00674

**Published:** 2022-12-08

**Authors:** Giovanni Cistaro, Mikhail Malakhov, Juan José Esteve-Paredes, Alejandro José Uría-Álvarez, Rui E. F. Silva, Fernando Martín, Juan José Palacios, Antonio Picón

**Affiliations:** † Departamento de Química, Universidad Autónoma de Madrid, 28049Madrid, Spain; ‡ Departamento de Física de la Materia Condensada, Universidad Autónoma de Madrid, 28049Madrid, Spain; § Instituto de Ciencia de Materiales de Madrid (ICMM), Consejo Superior de Investigaciones Científicas (CSIC), Sor Juana Inés de la Cruz 3, 28049Madrid, Spain; ∥ Instituto Madrileño de Estudios Avanzados en Nanociencia (IMDEA-Nanociencia), Cantoblanco, 28049Madrid, Spain; ⊥ Condensed Matter Physics Center (IFIMAC), Universidad Autónoma de Madrid, 28049Madrid, Spain; # Instituto Nicolás Cabrera, Universidad Autónoma de Madrid, 28049Madrid, Spain

## Abstract

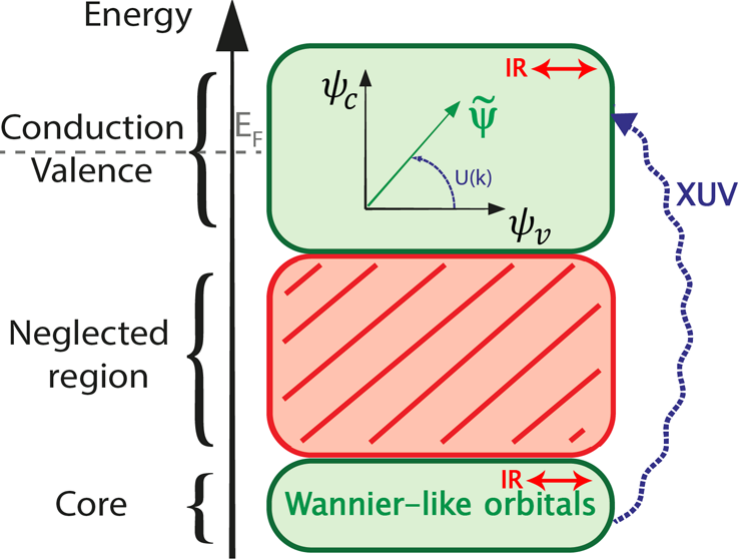

In this manuscript, we present a theoretical framework and its numerical implementation to simulate the out-of-equilibrium electron dynamics induced by the interaction of ultrashort laser pulses in condensed-matter systems. Our approach is based on evolving in real time the density matrix of the system in reciprocal space. It considers excitonic and nonperturbative light–matter interactions. We show some relevant examples that illustrate the efficiency and flexibility of the approach to describe realistic ultrafast spectroscopy experiments. Our approach is suitable for modeling the promising and emerging ultrafast studies at the attosecond time scale that aim at capturing the electron dynamics and the dynamical electron–electron correlations via X-ray absorption spectroscopy.

## I Introduction

Optical manipulation is the fastest technique to control and switch properties in a material. The advent of ultrashort laser pulses enables us to drive the system out of equilibrium and reach novel quantum phases with properties beyond the ones at equilibrium.^1^ Modifications of the topological phase,^2−6^ control of the valley pseudo-spin via optical resonant excitation,^7−9^ coherent light-driven currents,^10−12^ and light-induced insulator-to-conductor transitions^13−15^ are some promising applications within the out-of-equilibrium phenomena.

Significant progress has been achieved in recent years to implement time-resolved experiments for tracking and reading out transient state dynamics within the nonequilibrium system. Remarkably, it is nowadays possible to follow the electron dynamics in its natural time scale, i.e., in the attosecond time scale (10^–18^ s), and well before the lattice starts to respond to the external field. Attosecond transient absorption spectroscopy (ATAS) is a promising technique for tracking electron dynamics based on a pump–probe scheme, typically, the pump being a IR/mid-IR few-femtosecond pulse and the probe being an attosecond XUV/soft-X-ray attosecond pulse.^16^ The power of ATAS is that it combines attosecond temporal resolution with high energy resolution, much higher than that provided by photoelectron spectroscopy. ATAS has been successfully applied in different bulk and thin materials, from insulators to semimetals, to investigate carrier dynamics, phononic effects, and excitonic interactions.^17−24^

In this context, there is a natural need to simulate the transient state dynamics of condensed-matter systems under laser excitation, both for understanding the underlying mechanisms of out-of-equilibrium properties and for correlating the microscopic electron dynamics with the macroscopic measurements, observables such as current and absorption. The modeling of time-resolved experiments demands an additional complexity. In those, the probe pulse provides information of the out-of-equilibrium system for a specific time delay between the pump and probe pulse. However, the probe pulse must be included in the model, as it contributes to the dynamics and is directly linked to the measure of observables at particular time delays. Furthermore, the typical intensities of IR/mid-IR ultrashort pulses enable nonlinear interactions that must be accounted for in the model. Also, pump–probe schemes can be viewed as nonlinear schemes, as they require the absorption, at least, of two photons at two different times. Last but not least, in all nonmetallic two-dimensional materials known to date, the optical response is dominated by excitonic effects. This is, to a good extent, due to a suppressed screening of interactions in low dimensions, which facilitates the binding between electrons and holes.^25^ Excitons can be considered as quasiparticles composed of an electron–hole pair bound via Coulomb interaction. Hence, electron dynamics simulations must be able to describe the formation of excitons to properly describe the light–matter interaction. In this context, we aim at covering all of these demands, and we present in this manuscript a theoretical approach that allows us to simulate electron dynamics in realistic condensed-matter systems driven out of equilibrium as well as to model ultrafast/time-resolved spectroscopy experiments.

Density functional theory (DFT) is the workhorse of computational modeling for materials at equilibrium. However, out-of-equilibrium dynamics is beyond the scope of DFT, and there are three main alternatives. The first one is time-dependent DFT (TDDFT),^26−28^ which consists in solving a time-dependent Kohn–Sham (KS) equation. There are several TDDFT codes implemented in real time and in real space ideal for condensed-matter systems interacting with laser pulses, see for example, refs (29) and (30). To account for excitonic interactions in TDDFT, a long-range nonlocal exchange functional is needed, and the numerical implementation thus implies a high computational cost.^31^ The second one is based on many-body perturbation theory (MBPT). Starting from the Kohn–Sham DFT electronic structure, the well-known Bethe–Salpeter equation (BSE) is solved, and the energy and wave functions of excitons are obtained, the latter expressed as a superposition of single-particle excitations.^32,33^ BSE provides accurate energies, and it is ideal for spectroscopy calculations. However, BSE is not a time-domain framework; it cannot describe real-time nonequilibrium dynamics and ultrafast spectroscopy experiments. In recent years, there have been several theoretical approaches to extend BSE in the time domain.^34−36^ This consists of resolving the Kadanoff–Baym equations based on the nonequilibrium Green’s function theory.^37^

The third method is similar to solving the Kadanoff–Baym equations for the Green’s function, but starting instead from a second quantization formalism and evolving the reduced density matrix. Within this formalism, the well-known semiconductor Bloch equations can be derived,^38,39^ which evolve the density matrix of the system in reciprocal space. Our approach is based on this theoretical framework. We show how the equations of motion (EOM) for the density matrix can be efficiently implemented. We model relevant physical scenarios that illustrate the flexibility of our approach through either simple tight-binding (TB) models or within the Kohn–Sham DFT scheme, both with localized orbitals or Wannier basis, the description of excitonic effects in optical absorption spectra, and the feasibility to model ATAS using attosecond X-ray pulses both in two-dimensional (2D) and three-dimensional (3D) materials.

## II Theoretical Framework

In this section, we present the main theory for the evolution of the density matrix of a periodic system interacting with laser pulses. We detail the main approximations that are used in the numerical implementation.

### II.I Density Matrix

The many-body state of a periodic system can be represented in the second quantization formalism as
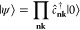
1in which |0⟩ represents vacuum, and the canonical operator *ĉ*_*n***k**_^†^ applying on the vacuum creates an electron in the state |*n***k**⟩. The quantum number **k** refers to the quasi-momentum, while *n* refers to the band (energy) level and spin. Typically, in the equilibrium, the state is populated up to a certain energy, the so-called Fermi energy or level. For a nonzero temperature, the equilibrium state should be represented by a statistical incoherent ensemble.

When a laser pulse interacts with the system at the equilibrium, the many-body state will evolve in time |ψ(*t*)⟩. To describe the system evolution that is driven out of equilibrium, one can use the reduced one-particle density matrix
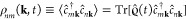
2where ϱ̂(*t*) ≡ |ψ(*t*)⟩⟨ψ(*t*)| is the evolving density operator, which can be easily generalized for a statistical incoherent ensemble. Note that, in general, one could use ⟨*ĉ*_*m***k**_^†^*ĉ*_*n***k**′_⟩, but we will show in the following section that the form given by eq 2 is sufficient to capture the evolution of the system. Note also that eq 2 is also valid for unpaired electron systems, suitable, therefore, to describe magnetic materials.

### II.II Many-Body Hamiltonian

The total Hamiltonian of a periodic system is expressed as
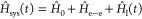
3in which *Ĥ*_0_ contains the noninteracting terms of all electrons, *Ĥ*_e–e_ accounts for the electron–electron interactions, and *Ĥ*_I_ is the laser–matter interaction. The latter term depends on the external electric field of the laser pulse and is then time-dependent. Other contributions that could arise from the lattice motion, such as phonon interactions, are neglected. This is justified because we aim at exploring very short time scales of the out-of-equilibrium system in the range of a few femtoseconds, in which the electron motion will be the relevant contribution. In particular, the different Hamiltonians are
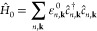
4
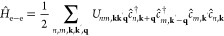
5
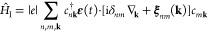
6where ε_*n*,**k**_^0^ is the energy dispersion of the band *n*, ξ_*nm*_ is the Berry connection, ε(*t*) is the electric field of the laser pulse, and *U*_*nm*,**kk**′*q*_ is the Coulomb interaction between two particles defined as *U*_*nm*,**kk**′*q*_ ≡ *W*_*n***k+q***m***k**′–**q**,*m***k**′*n***k**_, where
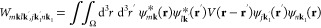
7*V*(**r** – **r**′) being the Coulomb energy between two particles and ψ_*n***k**_(**r**) ≡ ⟨**r**|*n***k**⟩.

The light–matter interaction term is written in the so-called length gauge *Ĥ*_*I*_(*t*) = |*e*|ε(*t*)·**r̂**, where **r̂** represents the position operator of the electrons in the system. This particular form is only valid in the dipole approximation when the size of the quantum system is smaller than the wavelength of the external electric field. For a unit cell, whose typical size is of few nanometers, this approximation is well justified for optical and IR wavelengths. However, it may be compromised for wavelengths lower than 1 nm, corresponding to photon energies larger than 1.24 keV (X-ray regime). For higher photon energies, higher multipole terms, such as the quadrupole term, can be considered and implemented within the present approach, but here, we restrict the light–matter interaction at the dipole approximation level. The position operator can be expressed as
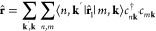
8where |*n*, **k**⟩ is the one-electron state defined as |*n*, **k**⟩ ≡ *ĉ*_*n*,**k**_|0⟩ and **r̂**_1_ is the position operator acting on one particle of the indistinguishable system. In general, for any basis satisfying the Bloch theorem, i.e., with the form ⟨**r**|*n*,**k**⟩ = ψ_*n*,**k**_(**r**) = e^i**k**·**r**^ *u*_*n*,**k**_(**r**), where *u*_*n*,**k**_(**r**) is a periodic function, the transition element is
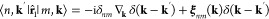
9in which the Berry connection is written as
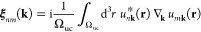
10The spatial integration is performed only over a unit cell Ω_uc_. This transition element gives rise to the used light–matter interaction Hamiltonian in the length gauge.

The numerical implementation of the *Ĥ*_e–e_ term, which also accounts for exciton–exciton interactions, requires a high computational effort. To reduce this effort, one can make the mean-field approximation that transforms this term in an effective single-particle operator
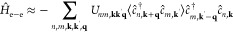
11In this approximation, one relies on the fact that the contribution *ĉ*_*n*_^†^*ĉ*_*m*_ in the Coulomb interaction is well described by the average ⟨*ĉ*_*n*_^†^*ĉ*_*m*_⟩, see ref (41). We assume excitations **q** = **k**′ – **k**, in which excitons do not carry momentum, i.e., ⟨*ĉ*_*m*,**k**_^†^*ĉ*_*n*,**k**′_⟩ = 0 if **k** ≠ **k**′, i.e.
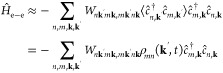
12The average term ⟨*ĉ*_*n*_^†^*ĉ*_*m*_⟩ corresponds exactly with the density matrix ρ_*mn*_(**k**, *t*) that we propagate in time. Hence, the Hamiltonian eq 12 requires to be computed at each time step. In real-space representation, the mean-field Hamiltonian eq 12 is nonlocal; see ref (42).

The fact that the full reduced density matrix is evolved in time, in contrast with TDDFT in which only diagonal terms are considered, permits the description of exciton effects via the Hamiltonian eq 12. TDDFT encounters difficulties finding a functional in which exchange and long-range effects are properly accounted, see ref (43) and references therein. In this work, we show that our formalism can produce correlated states and exciton effects at the same level as BSE. The main difference resides in the nondiagonal terms of the density matrix that represents excitations from the equilibrium analogously to the ansatz used in BSE. Note that before the time dynamics, no additional electron correlations are added besides the ones already included in the equilibrium system, and only when the system is driven out of equilibrium, electron–electron interactions play a role via eq 12.

### II.III Equations of Motion

Using the von Neumman equation for the evolution of the density operator, one can obtain the equations of motion (EOM) for the one-particle density matrix (eq 2) via iℏ∂ρ_*nm*_/∂*t* = Tr([*Ĥ*_sys_(*t*), ϱ̂(*t*)]*ĉ*_*m*_^†^*ĉ*_*n*_). In particular, the equations of motion can be reduced to
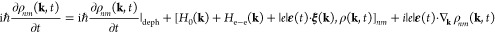
13where the matrix form is used, i.e.
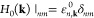

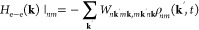

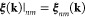
Note that the two light–matter interaction terms, which depend on the external electric laser field ε(*t*), are quite different. While the one with the Berry connections may induce interband and intraband transitions, the one with the gradient of the quasi-momentum **k** is purely intraband. The latter, which is related to the semiclassical electron propagation, is important for IR and THz fields and cannot be neglected for typical intensities between 10^9^ and 10^12^ W/cm^2^. Note also that electron–electron interactions make the EOM to be nonlinear with respect to the density matrix. All electron correlations beyond the mean-field approximation are included in the term iℏ∂ρ_*nm*_(**k**, *t*)/∂*t*|_deph_. Here, it is customary to include relaxation and dephasing effects arising from electron–electron interactions and electron–phonon couplings.

Note also that the formalism of the reduced density matrix accounts for the destruction of an electron in one state and the creation of an electron in another state, while the basis is the same during the dynamics. This formalism has a parallelism with time-dependent configuration interaction (CI) singles, in which the CI coefficients evolve in time because of the time-dependent nature of the Hamiltonian; see ref (44).

### II.IV Bloch Gauge

In the previous section, the equations of motion were introduced in the eigenstate basis, i.e., a basis in which the noninteracting Hamiltonian *Ĥ*_0_ is diagonal and satisfies *Ĥ*_0_|*n*, **k**⟩ = *Ĥ*_0_*ĉ*_*n*,**k**_|0⟩ = ε_*n*,**k**_^0^*ĉ*_*n*,**k**_|0⟩. However, it may be convenient to simulate the dynamics on another basis to reduce the computational cost of the equations of motion given by eq 13. In general, one can write a Bloch basis set as a linear combination of well-localized functions |α, **R**⟩
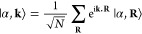
14Ideally, |α, **R**⟩ are functions that in real space decay exponentially (for instance, Gaussian orbitals or Wannier functions). This basis set satisfies Bloch’s theorem, even if it does not diagonalize the Hamiltonian *Ĥ*_0_. The sum of **R** goes over all unit cells of the system, and *N* represents the total number of unit cells. In this basis, note that the **k** dependence is in the imaginary exponential function of eq 14. Hence, the states are smooth functions of **k**. This has a clear advantage to simulate the dynamics, in which a less fine numerical **k** grid would be needed, in contrast with the eigenstate basis, which presents less smooth states and even singular points in the reciprocal space. In general, we can always express the eigenstates |*n*, **k**⟩ of our noninteracting Hamiltonian with respect to our Bloch basis, i.e.
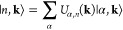
15or similarly *ĉ*_*n*,**k**_ = ∑_α_*U*_α,*n*_*ĉ*_α,**k**_, in which *ĉ*_α,**k**_ is an operator that creates an electron in our Bloch basis. Our change of basis is represented by a unitary matrix *U*|_α,*n*_ = *U*_α,*n*_, which may depend on the quasi-momentum. Vice-versa, one can write the Bloch basis in the eigenstate basis
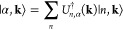
16Now, one can reformulate the equations of motion in the Bloch basis, defining the density matrix in this basis as ρ_αβ_^(*B*)^(**k**, *t*) ≡ ⟨*ĉ*_β**k**_^†^*ĉ*_α**k**_⟩
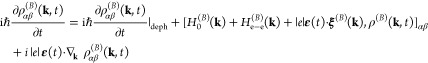
17Now, the different Hamiltonians are written on the Bloch basis, and they are connected with the eigenstate basis by
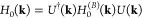

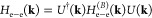

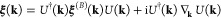
Note that the electron–electron transition elements *H*_e–e_ are computed at each time step through the density matrix at the eigenstate basis. In the Bloch basis, one may compute *H*_e–e_ by which first a basis change of the density matrix ρ(**k**, *t*) = *U*^†^(**k**)ρ^(*B*)^(**k**, *t*)*U*(**k**) and then a second basis change of the electron–electron Hamiltonian.

A gauge transformation can be considered as a unitary transformation such that *U*(**k**)|_*nm*_ = δ_*nm*_ e^–*i*φ_*m*_(**k**)^. Using the previous formalism, it is easy to check that the EOM are preserved under a gauge transformation.

### II.V Relaxation Effects

Electron correlations that are not considered within the mean-field Hamiltonian eq 12 and require a two-particle operator may be included in the term iℏ∂ρ_*nm*_(**k**, *t*)/∂*t*|_deph_ under certain approximations. This term includes not only electron–electron interactions that are important in X-ray interactions, such as Auger decay, but also electron–phonon scattering effects. These terms are challenging to compute, and some approximations are required to reduce their computational effort, but still, they describe the main underlying physics.

The decay of a core hole state is very fast, in the order of hundreds of attoseconds to a few femtoseconds, mainly triggered by Auger and fluorescence transitions. Ultrafast experiments with attosecond pulses are able to reach the soft-X-ray regime. At this regime, it is possible to access the relevant K-edge absorption lines of carbon, nitrogen, and oxygen. For light elements below neon, Auger transitions are the dominant ones in the core–hole decay. Auger transitions are described by a four-operator Coulomb interaction, in which an electron from a valence shell occupies the core hole, and the energy released from this transition is transferred to another valence electron that will be promoted into the free continuum. Typically, those transitions are interpreted as a state coupled to a continuum, which plays the role of a quantum bath. Within the so-called Markov or local approximation,^47^ the effects on the core–hole state can be reduced to a relaxation rate, i.e., iℏ∂ρ_*nm*_(**k**, *t*)/∂*t*|_deph_ ≈ – Γ_*nm*_^(ch)^ρ_*nm*_(**k**, *t*), where Γ_*nm*_^(ch)^ is a constant rate that is proportional to the inverse of the core–hole lifetime.

The effects of electron–phonon couplings, which play a role at longer time scales, can also be approximated using the electron–phonon Boltzmann scattering rate.^38^ To derive this scattering rate, the electron–phonon Hamiltonian needs to be considered, which enables to exchange energy and momentum between electrons and phonons. The scattering rates cannot then be described by relaxation or dephasing terms. Hence, the additional coupling creates new terms in the equations of motion. Considering phonons as a thermal bath and performing also the Markov approximation, one arrives at the electron–phonon Boltzmann scattering rate, which contains the sum of terms in the bath that depend on the product of the density matrix in **k** and **k** + **q**, where **q** is the momentum transfer to a phonon.

### II.VI Initial State

At zero temperature, all states below the Fermi energy level are assumed to be occupied, i.e., ρ_*nn*_(**k**, *t*_0_) = 1 if ε_*n*,**k**__0_ < ε_F_, being ε_F_ the Fermi energy. The electron–electron interaction gives rise then to an energy that depends on the initial equilibrium state. This reference energy can naturally produce a constant energy shift in our initial bands. To correct for this effect, this energy is subtracted from the total Hamiltonian, or what is the same, the electron–electron interaction can be computed as *H*_e–e_(**k**)|_*nm*_ = −∑_**k**′_*W*_*n***k**′*m***k**,*m***k**′*n***k**_(ρ_*nm*_(**k**′, *t*) – ρ_*nm*_(**k**′, *t*_0_)), in which the energy correlation arising from the equilibrium state is removed.

### II.VII Current

By analogy to classical electromagnetism, the current generated by an electron is given by the product of its charge and velocity **J** = −|*e*|**v**. Hence, one can calculate the derivative of the mean value of the position for an electron in a periodic system related to the current density as
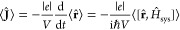
18where *V* is the volume of the system, *V* = *N*Ω_uc_, *N* is the total number of unit cells, and Ω_uc_ is the volume of a unit cell. For a two-dimensional material, the volume would be substituted by area, i.e., *V* = *NA*_uc_, where *A*_uc_ corresponds to the area of a unit cell. All electrons involved in the dynamics contribute to this formula. The position and the total Hamiltonian are single-particle operators, and after some algebra, the current can be cast as
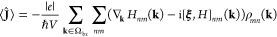
19in which the total Hamiltonian matrix is *H*(**k**) = *H*_0_(**k**) + *H*_e–e_(**k**) without the light–matter interaction Hamiltonian because it commutes with the position operator. Note also that the time dependence is both in the density matrix and in the excitonic interaction term. The previous formula has the same structure for any basis satisfying Bloch’s theorem. Therefore, current can be calculated in a Bloch-localized basis with no need to change to the eigenstate basis. The integration over the quasi-momentum should be restricted to the first Brillouin zone Ω_bz_.

The intraband current is defined in the eigenstate gauge as the part of the current that arises from the diagonal terms of the density matrix
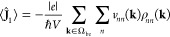
20where we define the velocity matrix as *v*_*nm*_(**k**) = ∇_**k**_*H*_*nm*_(**k**) – i[ξ, *H*]_*nm*_(**k**). It is easy to find the intraband current in the Bloch basis as
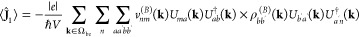
21On the other hand, knowing the total and the intraband current, the interband current is just obtained by subtraction ⟨**Ĵ**_2_⟩ = ⟨**Ĵ**⟩ – ⟨**Ĵ**_1_⟩.

### II.VIII Optical Absorption

The induced dipole density of the system, or polarization of the system, is given by
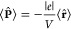
22where −|*e*|⟨**r̂**⟩ represents the dipole of all electrons and *V* represents the volume of the quantum system. By analyzing the gain and loss of a quantum system under a laser pulse, one can relate the Fourier transform of the polarization with the absorption coefficient as^44,46^
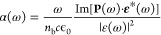
23where ϵ_0_ is the dielectric permittivity of the vacuum, *n*_b_ is the background refractive index, ε(ω) is the Fourier transform of the external electric field ε(*t*), and **P**(ω) ≡ ∫d*t *e^–iω*t*^ ⟨**P̂**⟩(*t*). Note that defining the Fourier transform of the current as **J**(ω) ≡ ∫d*t *e^–iω*t*^ ⟨**Ĵ**⟩(*t*), it is easy to check that *i*ω**P**(ω) = **J**(ω). The absorption coefficient has units of reciprocal length. For a two-dimensional material, because the volume is substituted by the area, the absorption becomes unitless. In this last case, the material can be interpreted as a zero-thickness layer between two dielectric media, and one has to take into account the Fresnel coefficients for the reflection and the transmission accordingly.^48^ The refractive index *n*_b_ is considered to slowly change with ω within the spectral bandwidth of the laser pulse. The absorption cross section is then σ(ω) = α(ω)/Π_c_, where Π_c_ is the number of unit cells per volume. Note that the formula for the absorption given by eq 23 is nonperturbative and is also valid for a broad bandwidth pulse, which is suitable to describe attosecond pulses that may have a bandwidth of more than 10 eV.

In the optical linear response regime, the polarization is proportional to the applied electric field *P*_*i*_(ω) = χ_*ij*_(ω)ε_*j*_(ω); χ_*ij*_ is the susceptibility at first order. For a particular polarization direction *i* (=*x*, *y*, *z*), the absorption coefficient is related to the imaginary part of the susceptibility by
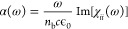
24If the coupling of the light with the material is weak, the absorption coefficient can be found in first-order time-dependent perturbation theory
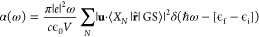
25where a set of final excited states given by single-particle excitations from valence (v) to conduction bands (c) is assumed, i.e., |*X*_*N*_⟩ = Σ_*vc***k**_*A*_cv_^(*N*)^(**k**)*ĉ*_c__†_*ĉ_v_*|GS⟩. The wavefunction *A*_*cv*_^(*N*)^(**k**) is typically found by solving the Bethe–Salpeter equation.^33^**u** is the polarization direction of the electric field. ϵ_f_ and ϵ_i_ are the energies of the final and initial states coupled by the laser light. In the absence of interactions, none of the electron–hole pairs are correlated, and the absorption in the linear response is given by the Kubo–Greenwood formula
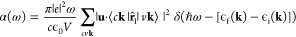
26In the linear regime, the optical conductivity σ_*ij*_ is related to the current *J*_*i*_(ω) = σ_*ij*_(ω)ε_*j*_(ω) and, consequently, to the susceptibility by *i*ω χ_*ij*_(ω) = σ_*ij*_(ω). More details are given in the Appendices A–C and in ref (52).

## III Numerical Implementation

In this section, we detail the numerical implementation for resolving the equations of motion for the density matrix of the system. These simulations require integration for hundreds of femtoseconds, implying an important computational effort, especially for three-dimensional materials that require a large grid. We design a code that resorts to both message passing interface (MPI) and open multi-processing (OMP) parallelization to speed up the simulations. In the following sections, we describe the main features.

### III.I Grid in the Reciprocal Space

The EOM for evolving the density matrix are defined in the reciprocal space. To reduce the computational effort, it is convenient to simulate the dynamics in the first Brillouin zone by also imposing periodic boundary conditions. In periodic systems, it is customary to work with Monkhorst–Pack grids^49^ that simplify the numerical implementation of boundary conditions for any given system with a particular spatial crystal symmetry. Any point in the reciprocal space can be written as **k** = *k*_*x*_**x** + *k*_*y*_**y** + *k*_*z*_**z** = *k*_1_**b**_1_ + *k*_2_**b**_2_ + *k*_3_**b**_3_, where (*k*_*x*_, *k*_*y*_, *k*_*z*_) are Cartesian coordinates in the direction of the canonical vectors {**x**, **y**, **z**}, while (*k*_1_, *k*_2_, *k*_3_) are crystal coordinates in the direction of the reciprocal lattice vectors {**b**_1_, **b**_2_, **b**_3_}. In crystal coordinates, it is easy to define the first Brillouin zone that spans from 0 to 1 in each coordinate. In each crystal direction, we take a discrete grid equally spaced. Any function that depends on the quasi-momentum *f*(**k**) is then represented by an array whose size is given by *N* = *N*_1_ × *N*_2_ × *N*_3_, where *N*_*i*_ is the total number of points in the corresponding crystal direction. Because of periodic conditions, the last point *k*_*N*_*i*_–1_ in a particular direction must be effectively the neighbor of the first one *k*_0_ when we calculate the gradient or the excitonic interactions.

Note that once we know any function in crystal coordinates, we can represent the function in Cartesian coordinates using the transformation
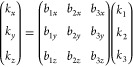
27where we define
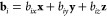
Note also that the discretization of the Brillouin zone can also be interpreted as the volume of the system, i.e., *V* = *N*_1_*N*_2_*N*_3_Ω_uc_.

Any integration with respect to the quasi-momentum in Cartesian coordinates is translated to crystal coordinates using the Jacobian determinant ∑_*k*_*x*_*k*_*y*_*k*_*z*__ → det(*M*)∑_*k*_1_*k*_2_*k*_3__, where *M* is the matrix of the transformation (eq 27).

### III.II Multidimensional Arrays

The density matrix and the different Hamiltonians depend on three parameters: the quasi-momentum **k** and the two band quantum numbers *m* and *n*. We represent them by multidimensional arrays with the structure *f*[*k*][*m*][*n*]. Here, *k* is an integer that is mapped to a reciprocal point **k** of the grid. In many loops, to calculate observables or compute the EOM, we run over these three parameters. As *k* is typically the parameter with more points, the external loop is always with *k*, while the internal loop is with *n*. This enables us to use multithreading in the *k* loop. We create special multidimensional arrays for *f*[*k*][*m*][*n*] whose data is aligned. This enables us to use autovectorization in the internal *n* loop, which does not compromise the multithreading in the external loop.

The Berry connections ξ(**k**) and the noninteracting Hamiltonian *H*_0_(**k**) can be calculated with any density functional theory or Hartree–Fock code that allows one to express them in a localized Bloch basis, as given in eq 14, such as CRYSTAL or SIESTA.^50,51^ Because in this basis they are smooth in **k**, we use a coarse grid to calculate them, and then we interpolate them to obtain a finer grid for resolving the time evolution, see ref (52) for more details of how to calculate the Berry connections. Similarly, we could use tight-binding models to provide the Berry connection and the noninteracting Hamiltonian via an analytical expression. Also, it is possible to calculate these elements using localized Wannier orbitals,^53,54^ which can be generated from codes such as Wannier90.^55^ In the next section, we show some examples in which we successfully implement different schemes based on Bloch, tight-binding, and Wannier basis.

### III.III Gradient in the EOM

The discretization of the reciprocal space in a Monkhorst–Pack grid requires a proper definition of the numerical gradient: the functions are not known at points spanned over the Cartesian axes but over nonorthogonal directions defined by the reciprocal lattice vectors.

A numerical implementation of the gradient in a Monkhorst–Pack grid has been extensively used in electronic structure calculations in equilibrium systems.^53^ The implemented gradient has a linear order precision.^56^ However, computing the equations of motion and resolving the nonequilibrium dynamics requires a higher precision to reach convergence.

**Figure 1:**
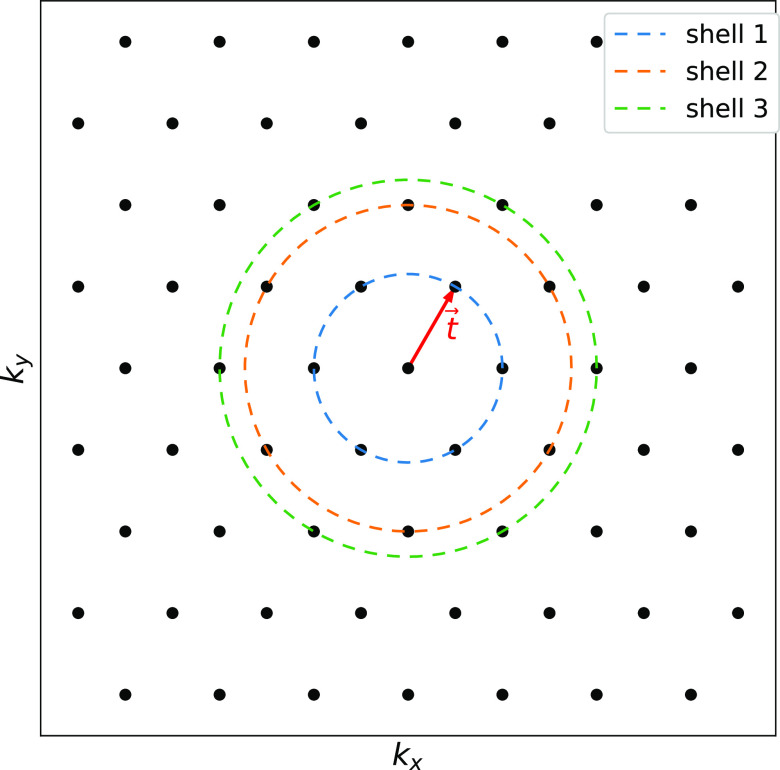
Monkhorst–Pack grid of the reciprocal space for a two-dimensional material with hexagonal symmetry. The calculated gradient at the point located at the origin depends on the neighbor points. Those are divided into shells, which are defined by the distance to the origin.

We extend the method used in ref (56) up to cubic order precision. The main difference lies on the constraints of the neighbor points to be satisfied. We define the vectors **t** that connect each **k** point of the grid with its closest neighbors. The **t** vectors are divided into shells and ordered by length; see Figure 1 for a two-dimensional hexagonal lattice. The gradient of a smooth function *f*(**k**) is calculated as
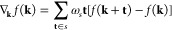
28where ω_*s*_ is the weight of a particular shell *s*. If the *s*th shell contains *M*_*s*_ vectors and the total number of shells is *N*_*s*_, the constraints to compute the gradient are
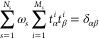
29
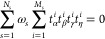
30Both equations need to be satisfied simultaneously. The Greek labels refer to the **t** vector components in the *x*, *y*, and *z* directions. Note that eqs 29 and 30 give rise to 6 and 15 different equations, so we have, in total, 21 independent equations. We can rewrite the constraints in a matrix form
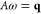
31where *A* is a matrix of dimensions 21 × *N*_*s*_, whose first six rows are
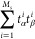
and the remaining 15 rows are
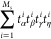
while ω and **q** are both vectors of length *N*_*s*_ and 21, respectively. The ω vector contains the weights of the shells, which are the ones to be calculated to be able to compute the gradient with the formula given by eq 28. The **q** vector contains the information on the right-hand side of constraints 29 and 30.

We construct the *A* matrix for each shell of increasing length, and we try to invert the system eq 31 using the Moore–Penrose pseudoinverse of *A*. The algorithm runs until the weights found using the pseudoinverse of *A*, i.e., ω = *A*^–1^**q**, satisfy eq 31.

### III.IV Parallelization of the **k** Grid

**Figure 2:**
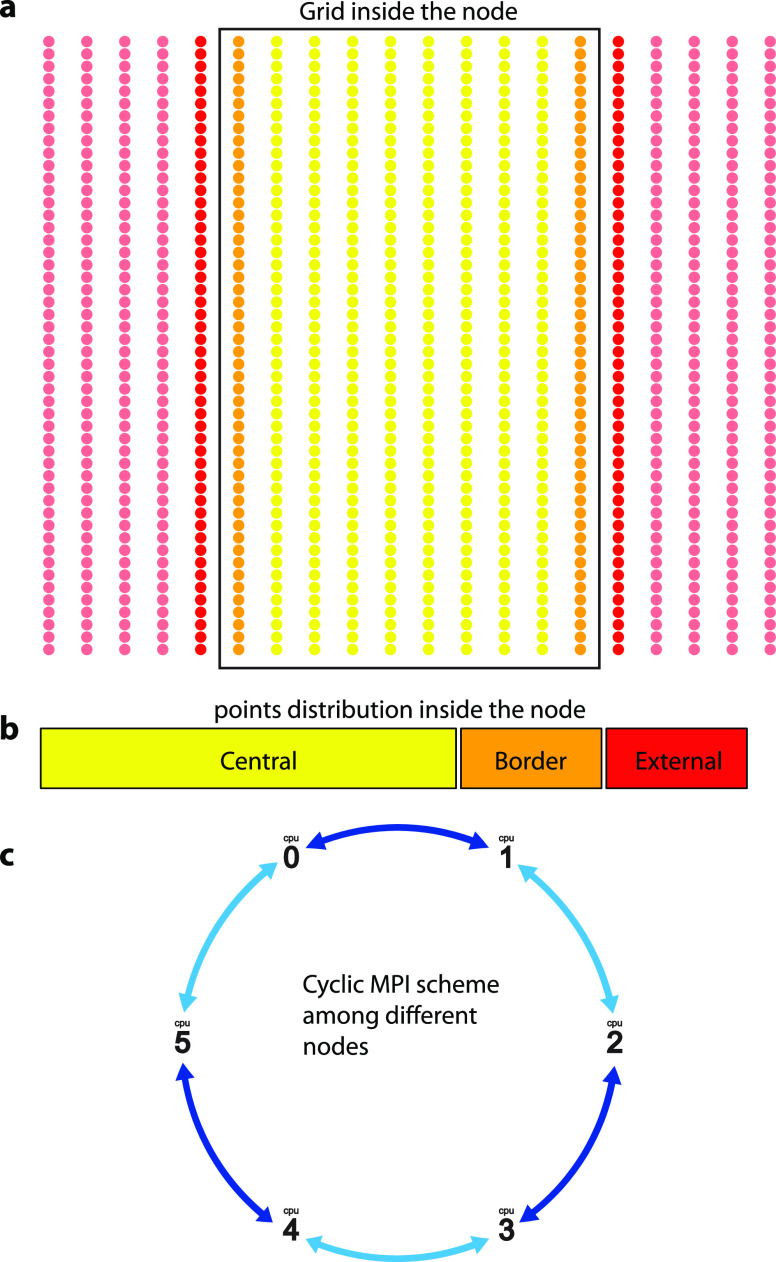
MPI communication scheme. (a) Two-dimensional grid in reciprocal space, in crystal coordinates, that is split in different nodes along the direction of one reciprocal lattice vector. (b) Inside a node, the **k** points are classified into central (yellow), border (orange), and external (red) points. The yellow part is propagated internally, the border part is passed to other nodes, and the external part is read from other nodes. (c) MPI communication scheme. Each node communicates with two different ones in a cyclic way, as defined in the picture.

The reciprocal space is divided into different nodes using MPI libraries; see Figure 2. Each node loads its corresponding initial parameters, such as noninteracting Hamiltonian and Berry connections, depending on its position in **k**. Hence, our multidimensional arrays *f*[*k*][*m*][*n*] can be distributed in different nodes and enable us to perform simulations that require a high demand of memory. In each node, multithreading and autovectorization are implemented, as previously described.

The gradient in **k** of the density matrix is part of the light–matter interaction term, and it is important for evolving the density matrix; see eq 13. The gradient of the noninteracting Hamiltonian is important to calculate the current, see eq 18, although this term is not time-dependent. The implementation of the gradient requires knowledge of the neighbor **k** points; see eq 28. This requires to set an MPI scheme to communicate this information among nodes, since each node is in charge of propagating only a part of the density matrix, there will be some **k** points whose neighborhood is not inside the same node. In particular, we identify the **k** points in three different categories; see Figure 2:

- Central points: in those points, the density matrix is evolved inside the node, and we do not need to transfer this information to other nodes.
- Border points: in those points, the density matrix is evolved inside the node, and we need to transfer this information to other nodes.
- External points: in those points, the density matrix is not evolved inside the node. The information of the density matrix at those points needs to be received for the propagation of other points.

To minimize the MPI communication, a good choice for splitting the reciprocal space is to cut in the direction defined by one of the reciprocal lattice vectors. This way, the external points will only lie on lines at the edges; see Figure 2a. This defines the different points of the grid inside the node as central, border, and external points. The communication is then cyclic for the nodes because the reciprocal space splitting is defined in a periodic direction; see Figure 2c.

At each step of the propagation, the density matrix located at the border and the external points are passed from one node to the ones within the neighborhood. This is the source of some overhead time. In the next section, we show some particular examples of two- and three-dimensional materials. The MPI scheme scales well for low number of nodes, approaching the  optimal law. The time propagation is implemented by a Runge–Kutta method of fourth order. Other time propagators are also implemented using Euler methods. One needs to check the time step to reach convergence. In the simulations presented in this work, convergence is reached with time steps around d*t* = 0.03 au (0.8 as). We have implemented then a dynamical time step to speed up the calculations as well as reach convergence.

### III.V Mean-Field Electron–Electron Interaction

In the mean-field approximation, the electron–electron interaction Hamiltonian becomes an additional time-dependent term to the noninteracting Hamiltonian; see eq 12. This involves a sum over the **k**-space of the density matrix times the Coulomb interaction *W*_*n***k**′*m***k**,*m***k**′*n***k**_, whose definition is a two-particle integral (eq 7). For Bloch wave functions ψ_*n*,**k**_(**r**) = e^i**k**·**r**^ *u*_*n*,**k**_(**r**), the Coulomb interaction can also be expressed as^35^
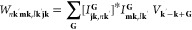
32where **G** is a sum over reciprocal lattice vectors, *V*_**k**_ is the Fourier transform of the Coulomb energy *V*(**r**), and
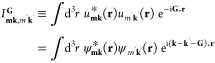
We can further expand the integral *I*_*m***k**,*m*′**k**′_^**G**^ using a localized Bloch basis (eq 14) via the unitary transformation of eq 15 and reduce the integral to
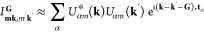
where we have used the approximation of very localized orbitals. Here, **t**_α_ is the position of the atom where the orbital is localized. Within this approximation, the electron–electron Hamiltonian in a Bloch basis *H*_e–e_^(*B*)^(**k**) is simplified as
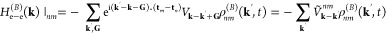
33From the numerical viewpoint, there is an advantage to work in a Bloch basis expanded in localized orbitals to avoid large grids for the reciprocal space. The expression given by eq 33 is extremely convenient to calculate the electron–electron interactions. Note that the density matrix evolves in time; therefore, this Hamiltonian also needs to be computed at each time step. Note that the effective potential *Ṽ*_**k**–**k**′__*nm*_ = ∑_**G**_e^i(**k**′–**k**–**G**)·(**t**_*m*_–**t**_*n*_)^*V*_**k**–**k**′+**G**_ is periodic in the reciprocal space. For the rest of the manuscript, we use *Ṽ*_**q**_^*nm*^ ≡ *Ṽ*_**q**_ to simplify the notation, but always keeping in mind that there is a phase factor that depends on the band indexes.

The dynamical mean-field interaction (eq 12) implies a high computational cost. First of all, for a grid with *N* points in the BZ, it requires to perform *N*^2^ operations at each time step. Second, the computation of this sum around **k** ≈ **k**′ requires more accurate evaluation due to the sharpness of the Coulomb interaction; see Appendix B. In the following sections, we discuss our numerical implementation to avoid the above-mentioned difficulties and exploit the same time parallelization resources.

We can always write the effective potential of eq 33 as *Ṽ*_**q**_ = *Ṽ*_**q**_^(*s*)^ + Δ*Ṽ*_**q**_, where *Ṽ*_**q**_^(*s*)^ is a smooth potential around **q** = 0 and Δ*Ṽ*_**q**_ contains the singularity at **q** = 0. To construct *Ṽ*_**q**_^(*s*)^, we change  in *V*_**q**_, where *q*_TF_ is a Thomas–Fermi screening parameter. This parameter makes the potential smooth around the origin, and in the limit of *q*_TF_ → 0, we recover then the singular potential. We therefore define Δ*Ṽ*_**q**_ = *Ṽ*_**q**_ – *Ṽ*_**q**_^(*s*)^, which is close to zero at long range and presents a singularity at close range. We can always choose a small *q*_TF_ such that *Ṽ*_**q**_^(*s*)^ reproduces well the initial potential in all **k**-space besides in a small area around *q* ≈ 0. In the following sections, we focus on two-dimensional systems, but the same procedure can be extended to three-dimensional ones. We expand the smooth function *Ṽ*_**q**_^(*s*)^ in Fourier series
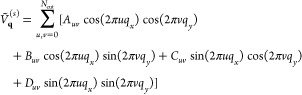
34where *A*_*uv*_, *B*_*uv*_, *C*_*uv*_, and *D*_*uv*_ are the Fourier coefficients and *N*_cut_ is the highest order harmonic that we take into account. The smooth potential (eq 34) is periodic, and it is relatively small at the boundaries of the first Brillouin zone. The dynamical mean-field interaction for the smooth potential is obtained by including eq 34 in eq 33
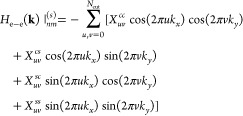
35*X*_*uv*_ are coefficients that do not depend on **k** and are expressed as
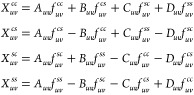
36where
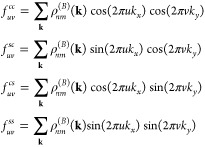
37The coefficients *A*_*uv*_, *B*_*uv*_, *C*_*uv*_, and *D*_*uv*_ can be computed at the beginning. However, the functions *f*_*uv*_ must be computed at each time step, as they depend on the density matrix. The sums over **k** in eqs 37 are performed in the first Brillouin zone. Note now that the number of operations to compute the mean-field interaction is *N* × 4*N*_cut_^2^. Hence, if the number of coefficients in the Fourier series is small in comparison with the number of grid points in the reciprocal space, which is the typical case, then this is already an advantage. Because the **k** grid is split in our parallelization scheme—see the previous section—each node requires to have information of the time-dependent *X*_*uv*_ coefficients to compute the mean-field energy (eq 35). For that, each node needs to compute its corresponding sum in eq 37 and communicate this number to the rest of the nodes to calculate the *X*_*uv*_ coefficients. Due to the small size of message communication, this approach is indeed efficient, even though the *X*_*uv*_ coefficients require to be computed at each time step.

**Figure 3:**
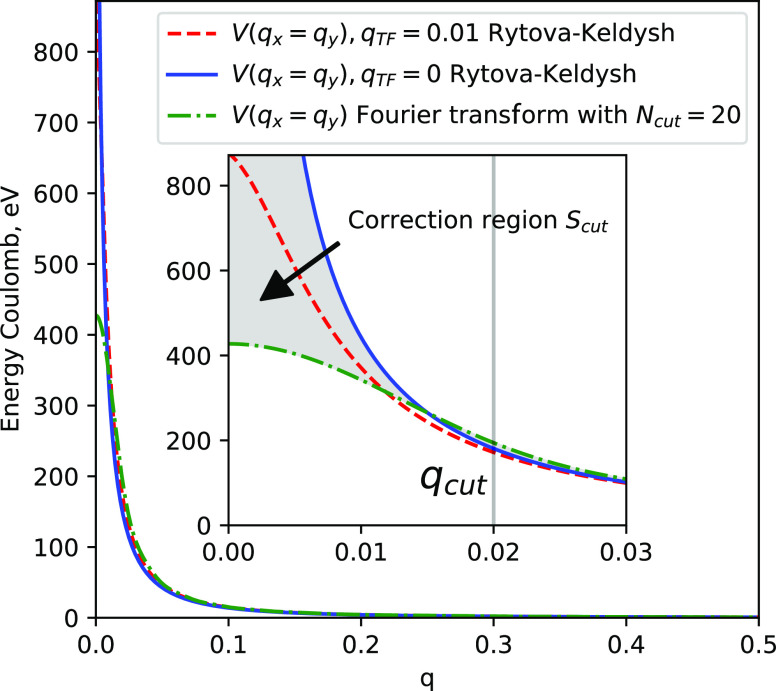
Comparison of exact Rytova–Keldysh potential *Ṽ*_**q**_ (blue line) and the same potential accounting for a Thomas–Fermi screening *q*_TF_, *Ṽ*_**q**_^(*s*)^ (red line). The expansion of the *Ṽ*_**q**_^(*s*)^ potential in Fourier series (green line), with *N*_cut_ = 20, is practically the same; the main difference is around the origin. The vertical black line shows the position of *q*_cut_. The inset is a zoom around the origin *q* = 0. The Rytova–Keldysh parameters are *A* = √3*a*^2^/2, *a* = 2.5 Å, *r*_0_ = 10 Å, and ϵ_1_ = ϵ_2_ = 1.

The exact interaction term can be computed as *H*_e–e_^(*B*)^(**k**)|_*nm*_ = *H*_e–e_(**k**)|_*nm*_^(*s*)^ + *X*_*nm*_^corr^(**k**, *t*), where *X*_*nm*_^corr^(**k**, *t*) is a correction at close range due to the Thomas–Fermi screening parameter
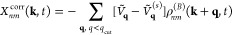
38Because the correction is mainly localized around **q** = 0, the sum over **q** is only meaningful around the origin; see for example, Figure 3 for a Rytova–Keldysh potential in a monolayer boron nitride. Hence, we expand the density matrix around the origin ρ_*nm*_(**k** + **q**, *t*) = ρ_*nm*_(**k**, *t*) + **q**·∂_**k**_ρ_*nm*_(**k**, *t*) + *O*(*q*_cut_^2^). Due to the symmetry of the potential, the linear terms vanish, and we can approximate the correction as
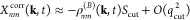
39where
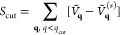
Note that the correction at a particular point is given by the density matrix at that same point times the factor *S*_cut_, which can be computed at the beginning and does not depend on time.

## IV Relevant Examples

In this section, we show three relevant examples for our real-time electron dynamics code. First is the calculation of light-induced current and optical/UV absorption in a monolayer boron nitride (hBN). These calculations are performed for both a TB model and a Kohn–Sham (KS) Hamiltonian obtained from CRYSTAL code, which uses Gaussian-type local orbitals as a basis. Second, we show the extension of these calculations when excitonic interactions are considered. Third, we show the calculation of an ATAS spectrum for realistic parameters in a pump–probe experiment in graphite.

In the Supporting Information, we show the inputs and outputs of these examples and provide some additional information of the structure of the code.

### IV.I Current and Optical/UV Absorption in hBN

**Figure 4:**
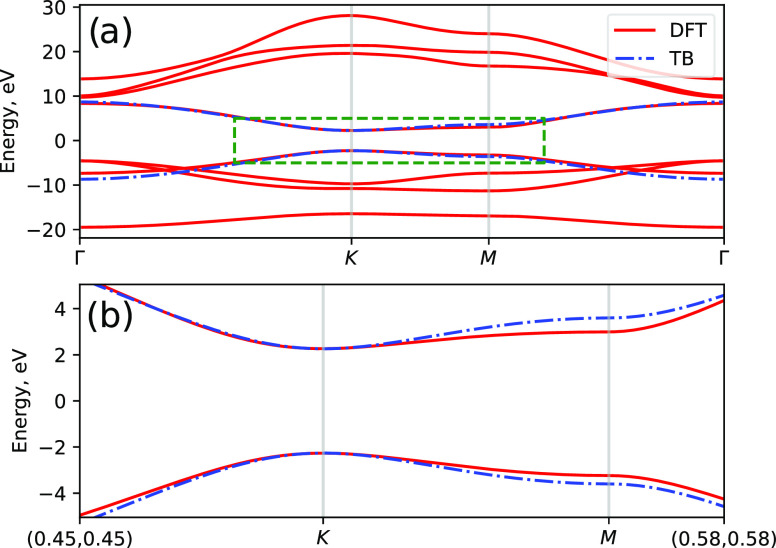
(a) Band structure of hBN along the path Γ–K–M−Γ. The TB parameters have been found by fitting the energy dispersion around the K point. (b) Zoom-in of the band energies around the K point marked by the green rectangle.

Monolayer boron nitride is a material with hexagonal symmetry and a large optical band gap of more than 4 eV. We first perform a DFT calculation within the LDA approximation using the CRYSTAL code.^50^ The unit cell has one atom of boron and nitrogen. A small number of Gaussian basis, one s- and three p-orbitals per atom, is enough to properly describe the energy structure around the band gap, see Figure 4. The band gap energy is 4.5 eV, lower, as expected, than the experimental value,^59^ but this is not essential to demonstrate the validity of our calculations. After calculating the Kohn–Sham electronic structure, we find the tight-binding model that fits the band structure around the K points; see Figure 4b.

**Figure 5:**
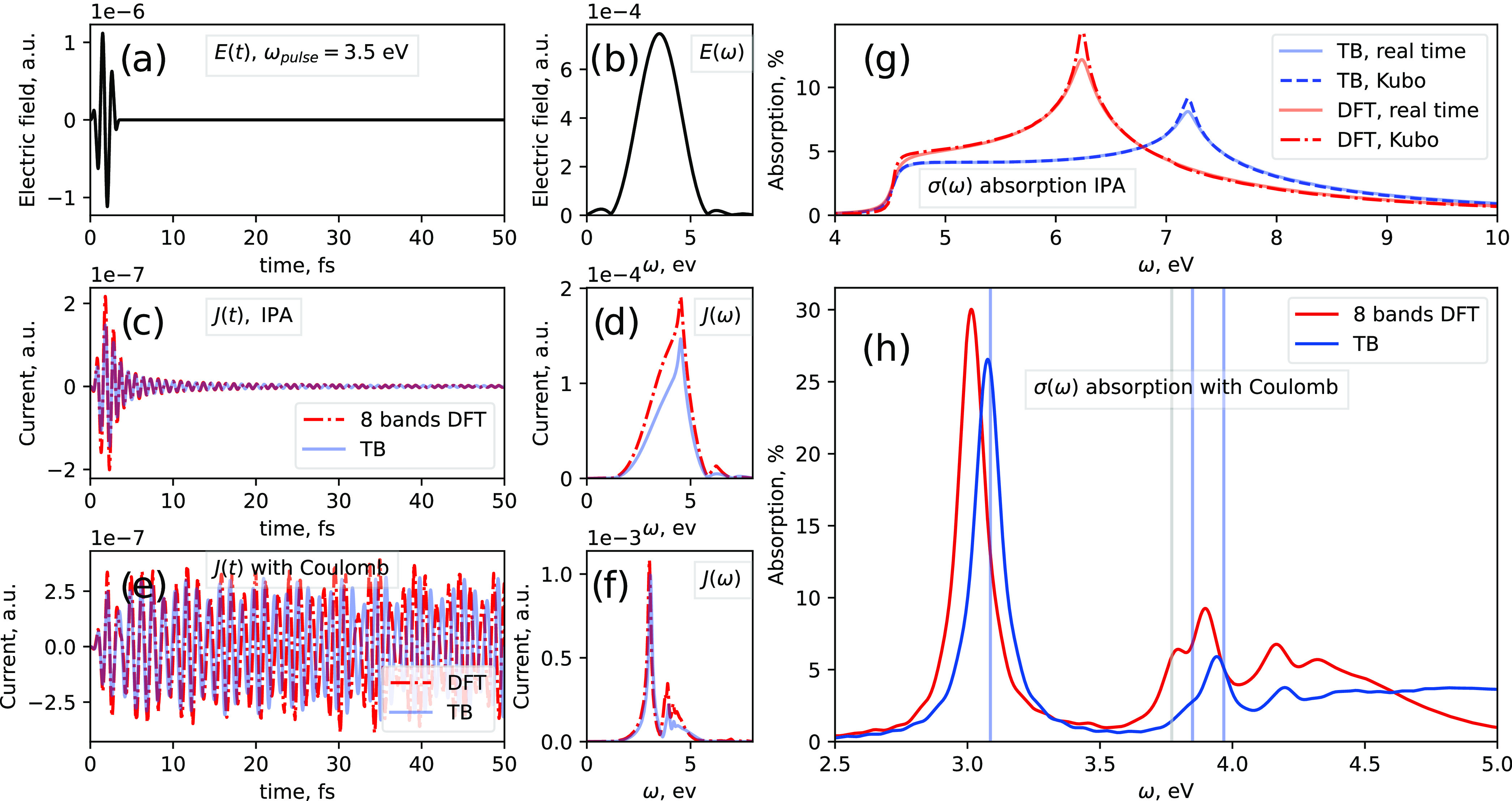
Current and absorption calculations in hBN. Panels (a) and (b) are the laser pulse in the time and frequency domain. Panels (c) and (e) are the light-induced current without (independent particle approximation, IPA) and with excitons. Red dash-dotted lines represent the results for an 8-band model obtained by DFT calculations, while solid blue lines are for a 2-band tight-binding model. Panels (d) and (f) are the corresponding Fourier transform. Panels (g) and (h) are the calculated absorption spectra without and with excitons. The real-time calculations are computed with eq 23 and compared with the first-order Kubo formula. The reciprocal space grid is *N* = 300 × 300 points, the number of terms in the Fourier series is *N*_cut_ = 20, and the time step is 1.2 as.

In this first example, we neglect electron–electron interactions described by *H*_e–e_(**k**) in eq 13. First, we calculate the light-induced current of the material by an ultrashort laser pulse. The time profile of the pulse is modeled by a sin^2^ envelope and a carrier wave of 3.5 eV frequency, as shown in Figure 5a. The pulse intensity is weak, 10^5^ W/cm^2^, to be at the first-order perturbation regime. The pulse is polarized along the Γ–M direction. The current is represented in Figure 5c. Note that even after the pulse, the current keeps oscillating and damps out after a few femtoseconds. The current for the TB model and the KS Hamiltonian show a very similar behavior. Second, we calculate the absorption of the laser pulse. The absorption is calculated within the bandwidth of the pulse, which it is around 4 eV centered at 3.5 eV; see Figure 5b. To calculate the absorption for other energies, such as for the highest energy point around 10 eV (the Γ point), we calculate the current for different pulse frequencies. Because the bandwidth is broad, few pulses are enough to cover the whole spectrum. The resulting absorption for the TB and KS Hamiltonians are shown in Figure 5g. The absorption increases after the band gap, and the lineshape is similar for both models. However, we find a clear difference at higher energies. The peak of the absorption is located at the M points, which are van Hove singularities. Those are located at different energies in the two models; see Figure 4b.

To demonstrate the validity of the absorption calculation, we compare the results using the Kubo–Greenwood formula (based on first-order perturbation theory) for a monochromatic laser given by eq 26. The Kubo formula is the standard method to obtain single-photon absorption spectra. The comparison is shown in Figure 5g. We observe that both procedures lead to similar results, apart from some smoothing in the absorption line of the real-time calculations due to the effects of a broad bandwidth pulse. This demonstrates the possibility to calculate optical/UV absorption spectra at the weak-intensity regime via real-time simulations. Furthermore, we could explore the nonlinear regime beyond first-order perturbation theory by increasing the intensity, or we could introduce a second pulse to model an ultrafast experiment, as we show in the last example for graphite.

The calculations were performed in Intel Xeon CPU E5-2630 type with 2.40 GHz. The size of the reciprocal space grid is *N* = 300 × 300 points. The time step is controlled dynamically and varies in the range of 1.2–0.3 during the simulation up to 80 fs. The 8-band DFT calculations required approximately 9 h on 2 nodes with 16 OMP threads each, while the TB calculations required approximately 1 h on one node with 16 OMP threads.

### IV.II Current and Optical/UV Absorption in hBN with Excitons

In this section, we investigate hBN using the same conditions as before, with the same laser parameters, but now turning on the excitonic (electron–electron) interactions.

First, we calculate the light-induced current; see Figure 5e. In comparison with our previous calculations, when interactions are not considered, the current is not damping out, and there is a clear current oscillation after the laser pulse. Comparing the Fourier transform of the current without and with interactions, see Figure 5d,f respectively, clear differences are appreciable with the TB and KS Hamiltonians showing a very similar behavior. Second, we calculate the absorption spectrum; see Figure 5h. The spectrum shows the characteristic exciton peaks below the band gap at 4.5 eV. The absorption quickly decreases after 5 eV. Interestingly, the TB model and KS Hamiltonian show very similar behavior.

We also compare the results with those obtained by solving the BSE equations for the TB model. The BSE is based on solving the time-independent Schrödinger equation to obtain the energies and wave functions of excitons. The BSE energies are given by vertical blue and gray lines in Figure 5h. We observe that the agreement is excellent for the blue lines. The gray lines correspond to dark excitons that are not excited by the laser pulse. This is in agreement with the previous analysis of excitons in hBN,^60^ in which two of the lowest-energy excitons are dark due to their symmetry.

The calculations were performed in Intel Xeon CPU type E5-2630 with 2.40 GHz. The size of the reciprocal space grid is *N* = 300 × 300 points, and the number of terms in the Fourier series expansion is *N*_cut_ = 20. The time step is controlled dynamically and varies in the range of 1.2–0.3 during the simulation up to 80 fs. The 8-band DFT calculations required approximately 11 h on 8 nodes with 16 OMP threads each, while the TB calculations required 11 h on one node with 16 OMP threads.

These calculations demonstrate the feasibility of the dynamical mean-field approximation to describe excitons, whose energies are the same as those obtained by solving BSE equations, in which the two-particle Hamiltonian is fully accounted. This opens the door to investigate excitonic interactions in real time for pump–probe and nonlinear schemes.

### IV.III ATAS in Graphite

We model an attosecond ultrafast spectroscopy experiment in a few-layer graphite. In particular, we model the changes of absorption of a X-ray attosecond pulse that goes through a material interacting with a mid-IR ultrashort pulse, i.e., we calculate the ATAS spectrum. These ultrafast experiments are currently feasible in advanced laser laboratories based on high-harmonic generation sources.^16^

**Figure 6:**
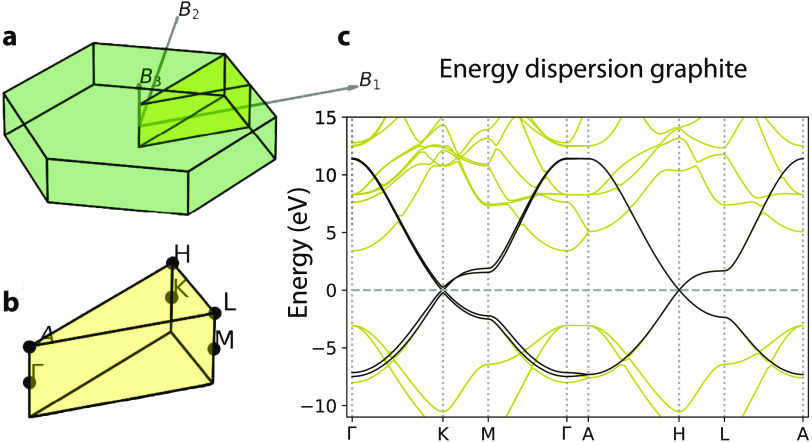
(a) First Brillouin zone of graphite and reciprocal lattice vectors. (b) Main points of the reciprocal space. (c) Energy dispersion along the path Γ–K–M−Γ–A–H–L–A.

For the simulations, we consider photon energies close to the resonant transitions between the 1s bands and the conduction bands, which are around 296 eV. The energy dispersion for graphite is shown in Figure 6 and is obtained using the Quantum Espresso code.^61^ In graphite, we have many monolayers of graphene that are separated by a distance of 3.35 Å. The unit cell contains four atoms of carbons. The first Brillouin zone is shown in Figure 6a. After calculating the electronic structure with Quantum Espresso, we create four Wannier orbitals using the Wannier90 code,^55^ which perfectly reproduce the energies of the four bands close to the Fermi level; see Figure 6c. To take into account the X-ray excitations, we also include the 1s orbitals of the four carbons in the unit cell, which are taken from electronic calculations of isolated carbon atoms.^62^ The four core bands are degenerate in energy. Because the 1s orbitals are well-localized, the corresponding energy-dispersion bands are flat. With a total of eight orbitals, we calculate the Berry connections that are used in our electron dynamics code.

We consider a weak X-ray pulse, intensity 10^9^ W/cm^2^, linearly polarized along the Γ–A direction, and a pulse duration of 80-as full width at half-maximum (FWHM). The bandwidth of the pulse is large enough to cover the whole energy range of the four bands close to the Fermi level, i.e., the bandwidth is larger than 18 eV. The mid-IR pulse has a wavelength of 3000 nm and a moderate intensity of 10^10^ W/cm^2^, enough intensity to promote electron carriers around the Fermi level. The laser is linearly polarized along the Γ–K direction, and it is very short, only 30 fs (around three cycles). The core–hole decay of the carbon is dominated by Auger processes. We include a core–hole decay of width Γ_ch_ = 0.108 eV, corresponding to a lifetime of 6.1 fs, the expected lifetime of the 1s vacancies at a carbon atom. The bands are occupied below the Fermi level, i.e., *T* = 0 distribution.

**Figure 7:**
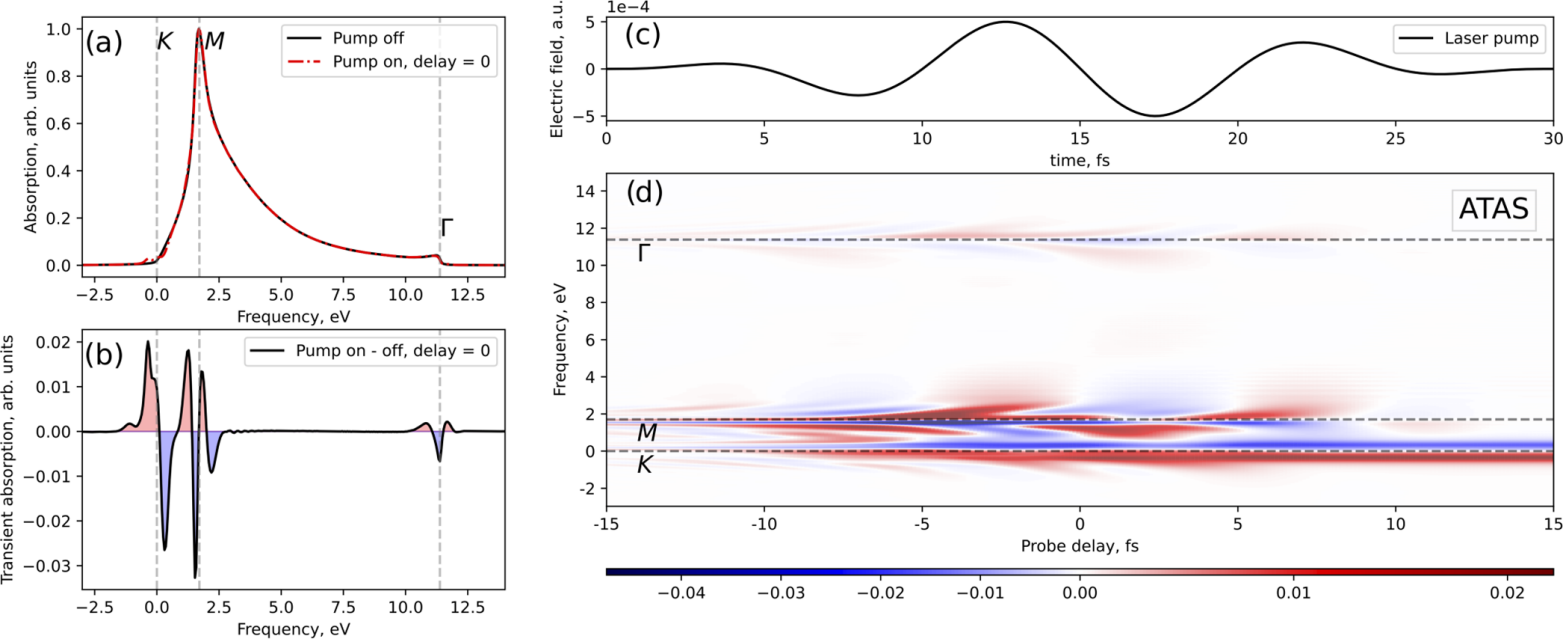
X-ray attosecond transient absorption in graphite. (a) Calculated absorption spectrum with (red line) and without (black line) the IR pulse in the case of zero delay. (b) Difference between the two spectra, with and without the IR pulse that shows changes due to the transient dynamics. The time delay between the two pulses is 0 fs (the maximum points of the pulse envelopes are overlapping). (c) IR pulse in time. (d) ATAS spectrum. Negative time delays correspond when the attosecond pulse arrives first.

The calculated X-ray absorption spectrum is shown in Figure 7a for a 0 fs time delay, i.e., when the peaks of the envelopes of both pulses are overlapping. The zero in the energy scale corresponds to the transition from the 1s band to the Fermi level. We show the calculated absorption without the mid-IR pulse; see the black line. We repeat the simulation, but in the presence of the mid-IR laser pulse, and we observe clear changes in the absorption; see the red line. By taking the difference, we obtain the transient absorption spectrum with changes that are mainly localized around van Hove singularities; see Figure 7b. This is similar to what has been reported in graphene.^45^ We obtain the ATAS spectrum by repeating the same transient absorption calculations for different time delays between the X-ray attosecond and mid-IR pulse; see Figure 7d. For reference, we show the mid-IR pulse in time in Figure 7c. The changes around the Fermi level (such as K and H points) are due to the promotion of electrons from the valence to the conduction, which affects the excitation process itself due to Pauli exclusion. The other two features, around 2 and 12 eV (where the M,L and Γ,A points are located, respectively), are related to the coherent laser-driven dynamics of electrons in the conduction band promoted from core bands.^45^ We should remark here the importance of treating both pump and probe pulses on an equal footing to obtain the transient absorption spectrum. With the current technology,^24^ it will be possible to carry out an ultrafast experiment similar to the one modeled in this work.

The calculations were performed in Intel Xeon Platinum 8160 with 2.10 GHz. The size of the reciprocal space grid is *N* = 200 × 200 × 10 points. The time step is 2.4 during the simulation up to 80 fs. The calculations for each time delay took 12 h using 4 CPUs with 48 OMP threads.

In summary, these calculations demonstrate the possibility to use Wannier orbitals to compute real-time simulations as well as to describe ultrafast spectroscopy studies.

## V Conclusions

We have presented a theoretical framework and numerical implementation to simulate the out-of-equilibrium electron dynamics that arise when a condensed-matter system is irradiated by ultrashort laser pulses. The approach is based on real-time simulations in the first Brillouin zone by imposing periodic boundary conditions. We solve the equation of motion of the reduced one-electron density matrix of the system and calculate observable expectation values in the time domain, such as light-induced current and polarization in the medium. The approach is suitable to account for: (i) light–matter interactions for different laser pulses, (ii) a wide range of photon energies within the mid-IR to the soft-X-ray regime, and (iii) excitonic interactions within the dynamical mean-field approximation, and (iv) work with any localized Bloch basis. Furthermore, the approach can work with the electronic structure previously calculated with a DFT code. We show the robustness of the code to work with local orbitals and Wannier basis from CRYSTAL and Wannier90 calculations. As a relevant example, we show the calculations of the current and optical/UV spectrum for hBN with and without excitons via real-time simulations. Also, we model a realistic absorption pump–probe experiment with a mid-IR laser and an attosecond X-ray pulse in graphite.

Due to the flexibility of our approach to use Bloch states on a local orbital basis previously calculated from DFT calculations, we envision the possibility to describe the light-induced response in functional materials of current interest, such as the photogalvanic effect and the HHG emission, and also to describe novel ultrafast spectroscopy studies.

## Appendix A

### X-ray Interactions

In time-resolved experiments, the system is brought out of equilibrium by a laser pulse, the so-called pump pulse, and the dynamics are studied by a second laser pulse, the so-called probe pulse. To calculate the observables for the probe pulse, one needs to calculate the transient electron dynamics induced by both pulses. However, most of the time, the probe pulse has a photon energy (frequency) much higher than the pump pulse. In attosecond science, typically, the pump frequency is in the IR/mid-IR range, while the probe frequency is in the XUV/soft-X-ray range. Simulating the electron dynamics of both pulses is a challenging task because one needs a fine resolution in time to properly describe the probe pulse effects, while the pump pulse forces a long integration in time. To reduce this effort, one may perform the rotating-wave approximation (RWA) on the probe interactions. The external electric field is split in ε(*t*) = ε_*p*_(*t*) + ε_*x*_(*t*), where ε_*p*_(*t*) and ε_*x*_(*t*) correspond to the pump and probe electric field, respectively. The probe pulse is described as ε_*x*_(*t*) = **g**_*x*_(*t*) cos ω_*x*_*t*, where **g**_*x*_(*t*) is a slowly variant envelope function and ω_*x*_ is the central frequency. The probe interactions involve a core band and a valence/conduction band, and the corresponding off-diagonal term of the density matrix oscillates then very rapidly, with frequencies of the order of the central frequency ω_*x*_. Writing the off-diagonal term of the density matrix as ρ_*nn*_c__ = e^–iω_*x*_*t*^ ρ̃_*nn*_c__, where *n*_c_ and *n* labels refer to the core band and a valence/conduction band, respectively, enables us to separate the fast- from the slowly variant changes in time. Including this transformation in the EOM, eq 13, and performing the RWA, i.e., neglecting the fast terms in time, one obtains^44^

40

where the off-diagonal terms involving a core band *n*_c_ and a valence/conduction band *n* are cast as

otherwise

Note that also the excitonic interaction accounts for the slow-variant terms in the off-diagonal terms involving core electrons.

## Appendix B

### Coulomb Potentials

The Fourier transform of the Coulomb energy between electrons *V*(*r*) = *e*^2^/4πϵ_0_*r* is given by^38^

41

for a 3D and 2D material. However, in the case of two-dimensional materials, due to the dielectric effects of the environment and screening, it is more convenient to use the so-called Rytova–Keldysh potential^57,58^

42

which is a 2D electrostatic potential derived for a thin layer embedded between two dielectrics. *H*_0_ and *Y*_0_ are the Struve function and the Bessel function of the second kind. The screening length is *r*_0_ = *d*ϵ_m_/(ϵ_1_ + ϵ_2_), where *d* is the thickness of the material and ϵ_m_, ϵ_1_ and ϵ_2_ are the dielectric constants of the material, the top dielectric medium, and the bottom dielectric medium, respectively. The Fourier transform of the Rytova–Keldysh potential is given by

43

In the case of a free-standing monolayer, ϵ_1_ = ϵ_2_ = 1.

## Appendix C

### Absorption and Conductivity in Linear Response

#### Linear Response Using Time-Dependent Perturbation Theory

The absorption calculated using eq 23 can be compared with the formula of the absorption obtained in first-order linear response approximation as long as we are in the weak-intensity regime. We consider the absorption of a photon of ℏω energy via the energetic transition Γ_*f*→*i*_, with Γ_*f*→*i*_ the number of transitions per unit time per unit volume, normalized to a linearly polarized incident flux *I*(ω) = 2*c*ϵ_0_|ε(ω)|^2^ as

44

Here, ε(ω) refers to the Fourier transform of the electric field with positive frequencies. All transition processes from the initial state are considered here. The transition rate is now worked out using time-dependent perturbation theory (similarly to using Fermi’s Golden Rule^40^)

45

where ϵ_f_ and ϵ_i_ correspond to the unperturbed energies of the final and initial eigenstates, respectively. Inserting the Fourier transform of the electric field, defining ϵ_f_ = ℏω_f_ and ϵ_i_ = ℏω_i_, and performing the integration in time

46

Assuming that the main contribution of the integral is for frequencies very close to the transition resonance, then it is a good approximation to reduce the square modulus to the integrand,^40^ i.e.

47

In the limit *t* → ∞, the above oscillating function tends to 2πℏ*t*δ(ℏω – [ϵ_f_ – ϵ_i_]). After the time derivative and considering only the positive frequencies that satisfy the energy conservation

48

Hence, the absorption, depending on the frequency, is cast as

49

where **u** is a unitary vector indicating the polarization direction of the incident field. In linear response approximation, the frequency-dependent polarization is linked to the conductivity as **P**(ω)|_*k*_ = −iω^–1^**J**(ω)|_*k*_ = −iω^–1^σ_*kj*_(ω)ε(ω)|_*j*_, with σ_*kj*_ the two-rank conductivity tensor. In linear response, the absorption is related to the first-order susceptibility χ as

50

where **P**(ω)|_*k*_ = χ_*kj*_(ω)ε(ω)|_*j*_ and **u** refer to the polarization direction of the field. The conductivity and the susceptibility are related then by σ_*kj*_(ω) = iωχ_*kj*_(ω). Using this relation and the absorption formula given by eq 49, the real part of the optical conductivity is

51

#### Conductivity: Single-Particle Approximation and Excitons

The above formula must be particularized for a set of final excited states. Considering electron–hole correlations, exciton states are written as |*X*_*N*_ = Σ_*vc***k**_*A*_*cv*_^(*N*)^(**k**)*ĉ*_*c*_^†^*ĉ*_*v*_|GS⟩, where one uses a basis of single-particle excitations states from valence (v) to conduction bands (c). The wavefunction *A*_*cv*_^(*N*)^(*k*) is typically found by solving the Bethe–Salpeter equation.^33^*N* is the number of exciton states. In our case, this number is related to the number of points in the reciprocal space. The optical conductivity then reads^63^

52

The matrix elements can be computed using the single-particle states as ⟨*X*_*N*_|**r̂**|GS = Σ_*cv*k_*A*_*cv*_^(*N*)^(**k**)⟨*c***k**|**r̂**_1_|*v***k**⟩. In the absence of interactions, none of the electron–hole pairs are correlated, and the optical conductivity formula in linear response is reduced to the well-known Kubo–Greenwood formula

53

## Supporting Information Available

Structure of the code; structure of the input; structure of the output; and examples (PDF)
