# Supplementary material for: Theoretical Approach for Electron Dynamics and Ultrafast Spectroscopy (EDUS)
Source: J Chem Theory Comput. 2022 Dec 8;19(1):333–48. doi: 10.1021/acs.jctc.2c00674 (PMC9835834; doi:10.1021/acs.jctc.2c00674)
Supplement: Supplementary file 1 — ct2c00674_si_001.pdf [file ct2c00674_si_001.pdf]

Supplementary Information

Content:
1) Structure of the code
2) Structure of the input
3) Structure of the output
4) Examples

Additional data at the external repository DOI: 10.21950/KILVLZ

1) Structure of the code

The main core of the code, where the optimization is required, is the time propagation for resolving the equations of motion. In the following, we explain the main structure of our implemented code and we connect each part with the numerical methodology explained in the main text.

The structure of the code follows:

i) Creation of the basic variables and reading of the input. In the input, one needs to define which type of electronic structure input we have. If it is a tight-binding model, one can easily implement the analytical formulas in the code. If the calculations are performed with a secondary code, such as CRYSTAL, then one needs to provide the energy dispersion, the Berry connection, and the general information of the reciprocal grid.

ii) After reading the electronic structure data, one needs to divide the reciprocal space as explained in section III.D. of the maintext for the MPI parallelization. In this repository is shown the part of the code that performs this MPI splitting.

iii) Allocate the electronic structure data in multidimensional arrays as explained in section III.B. of the maintext. This simplifies the use of the OpenMP and MPI libraries.

iv) Define Coulomb energy, depending on the system, as Eqs. (35-37), and expand the Coulomb interaction in Fourier series as Eq. (38). Also, calculate the correction for the Fourier series around the singularity by using Eq. (43), see more details of the implementation in section III.E. of the maintext.

v) Resolve the equations of motion, see Eq. (13) of the maintext, by using a time-propagator algorithm such as Runge-Kutta. For the time propagator, it is important to use the definition of the gradient as in Eq. (29), which preserves the symmetry of the crystal. For the implementation of the gradient one needs to define the shells and the

weights for each shell as explained in section III.C. This algorithm provides a high accurate calculation of the gradient and reduces significantly the numerical error in the time propagation.

vi) For each time step, one needs to calculate the electron-electron interaction in the dynamical mean-field approximation, see Eq. (34) and (39). Because that terms involve a sum over all points of the BZ, and the grid is distributed among different nodes, then each node will perform the corresponding part of the sum and the total sum needs to be delivered to all nodes.

vii) For each time step, one needs to calculate the current as in Eq. (20), which is easy to implement. Here, the gradient of the Hamiltonian can also be calculated by using Eq. (29). In the repository, some examples of inputs and outputs are provided.

2) Structure of the input

The input to the program is located in the file named “input.txt”. The structure of the input is grouped in blocks. Each block corresponds to different part of the code and it will change specific parameters. The order of blocks and lines does not matter. In the repository, one can find some examples. In the following we provide an explanation of the different blocks of the inputs:

i. Block tdse{} provides information about the time propagator and the model we use. We specify if we read band structure from a file, for example from a calculation from CRYSTAL or Wannier90, or use tight-binding model, which the formula of the electronic structure is already implemented. For tightbinding, we have to specify dielectric gap and hopping parameters. In this block, we also specify the time step for the time evolution and the final time, i.e. when the time evolution stops. Important flags: tightbinding, CRYSTAL, wannier, and dt. See examples in the repository.

ii. Block Coulomb{} is needed to define Coulomb interaction. If we do not have it, program will start in independent particle approximation (IPA) mode. Here qTF is a Thomas-Fermi screening parameter that is explained in section III.E. Ncut is number of terms in Fourier series in eq. (38). Variable epsilon_static is a constant $(\epsilon_1 + \epsilon_2)A$ from formula (37). Also, we need a flag Rytova_Keldysh to define our potential type. Please see examples in the repository.

iii. Block nkPT{} is needed to define the grid for 3 coordinates. Our 2D sample should lie in a second and third dimension.

iv. Decoherence{} contains information of the dephasing and loss terms is important for calculations with core holes. and it contain corehole decay parameter

v. laserpump{} block allows us to choose one of the laser pulses, typically the pump pulse. In our manuscript for example corresponds to the UV laser pulse. One defines after the name the envelope of the pulse. The most convenient ones are sin2 or gaussian. One can also define the number of cycles or pulse duration, the wavelength or photon energy, and the intensity laser pulse. The polarization can be chosen in 3 directions, we should specify from 0 to 1 the proportion of intensity in each direction.

vi. laserprobe{} block is to include a second laser, typically the probe pulse. This one is used to perform ultrafast/pump-probe simulations. Analogously to the laserpump block, one can specify the envelope, pulse duration, wavelength, intensity, and polarization of the laser probe pulse. An additional parameter is the time delay, which corresponds to the time delay between the maximum intensity of the envelopes of the pump and probe pulses.

vii. observables{} block defines which observables and quantities the program needs to calculate and print. In case of this article we need flags Current and Tabs which prints current and transient absorption respectively. We do not print results every time step of the propagation and it_resolution defines how many steps we have between neighbor savepoints. In particular, “it_resolution 2” means that we print result every second timestep.

3) Structure of the output

When running the code, several output files are printed, the most important ones are:

i. All intermediate results are printed in “output.log” file. There we can recheck the input parameters, such as laser properties, unit cell, model used, etc. and follow in which time step the calculation is.

ii. “EF.txt” provides the information of the electric field of the pump laser pulse. The file contains 4th columns: the first column corresponds to the time (in femtoseconds), and three remaining columns of the value of the electric field in the three different directions (x-,y-, and z- direction).

iii. “Losses.txt” provides information of the band population in the eigenstate basis in time, in the range of [0,1], being 0 no occupied and 1 fully occupied. The first column corresponds to the time (in femtoseconds), and remaining columns correspond to the band population, one column for each band.

iv. Files “J1.txt” and “J2.txt” contain current components from eq. (20), i.e. “J1.txt” contains the current coming from the gradient of the Hamiltonian, while “J2.txt” contains the current coming from the commutator of the Berry connection with the Hamiltonian. The first column of the files corresponds to the time (in fs). Then the remaining columns correspond to the current in time in the x-,y-, and z- direction. Note that J1 has an additional column for each direction corresponding to the imaginary part of the value. This is just to double check that J1 is real and the imaginary part is low enough to be considered 0 within the numerical error. Remember that the current is calculated from the density matrix that is a complex variable, but an observable should be real.

v. The “TransientAbs.txt” file contains information for probe pulse absorption. The first column corresponds to time (in fs). Columns 2-4 are intensities of the probe pulse in x-,y-, and z-directions. Columns 5,7,9 are real and columns 6,8,10 are imaginary part of the polarization of the system, see eq. (23), which is needed to calculate the ATAS.

4) Examples

One can find three main examples of the calculations performed in this manuscript; i) for monolayer BN (hBN) with excitons, when the electronic structure is calculated by a TB model, ii) for monolayer BN (hBN) with excitons, when the electronic structure is calculated with the CRYSTAL DFT code, and iii) for graphite for ATAS calculations, when the electronic structure is calculated with a Wannier basis from the Wannier90 code.

i. For modeling the optical/UV absorption of hBN with excitons, we use a TB model whose formulas for the hamiltonian and the Berry connections are already implemented within the code. In the example, a time dynamics calculation is performed with 3 bands; 1 core band, 1 valence band, and 1 conduction band. Only a pump pulse is used by using a zero intensity in the laserprobe input. The pump pulse frequency (4 eV) is close to the bandgap energy. In the input, “gap” label set the bandgap energy, and “t1” label set the first order hopping, both in electronvolts units.

ii. For modeling the optical/UV absorption of hBN with excitons, we use a localized basis obtained from the CRYSTAL code. The code requires additional inputs to read the electronic structure. In particular, four files: the “kmesh_data.dat” is a file that contains the information of the crystal and the information of the grid in k-space used for the electronic structure calculations, the “hk_orthonormal.dat” is the Hamiltonian in the used k-space grid, and the “bc_betabetap_real.dat” and “bc_betabetap_imag.dat” are the real and imaginary part of the

berry connections in the used k-space grid. In the example, a time dynamics calculation is performed with 8 bands; 4 valence bands and 4 conduction bands. Only a pump pulse is used by using a zero intensity in the laserprobe input. The pump pulse frequency is close to the bandgap energy.

iii. For modeling the ultrafast scheme in graphite and to calculate the ATAS spectrum, we use a Wannier basis obtained from the Wannier90 code. The code requires additional inputs to read the electronic structure. In particular, four files: the "graphite_tb.dat" that is a file generated by Wannier90, the "dipole_cc.dat" corresponding to the Berry connections between core and core orbitals, the "dipole_cv.dat" corresponding to the Berry connections between core and valence/conduction orbitals, and the "Hcc.dat" corresponding to the energies of the core orbitals. In the example, a time dynamics calculation is performed with 8 bands; 4 core bands, 2 valence bands, and 2 conduction bands. Two pulses are used. The pump pulse is a mid-IR pulse, and the probe pulse is an X-ray pulse whose frequency is tuned to cover the valence and the conduction band. The example is for a specific time delay, it can be changed in order to modify the time delay between the pulses keeping all the other parameters fixed.
